# Phonon Properties and Lattice Dynamics of Two- and Tri-Layered Lead Iodide Perovskites Comprising Butylammonium and Methylammonium Cations—Temperature-Dependent Raman Studies

**DOI:** 10.3390/ma17112503

**Published:** 2024-05-22

**Authors:** Mirosław Mączka, Szymon Smółka, Maciej Ptak

**Affiliations:** Institute of Low Temperature and Structure Research, Polish Academy of Sciences, Okólna 2 str., 50-422 Wroclaw, Poland; s.smolka@intibs.pl (S.S.); m.ptak@intibs.pl (M.P.)

**Keywords:** hybrid organic–inorganic perovskites, lead halides, Raman, lattice dynamics, phase transition

## Abstract

Hybrid lead iodide perovskites are promising photovoltaic and light-emitting materials. Extant literature data on the key optoelectronic and luminescent properties of hybrid perovskites indicate that these properties are affected by electron–phonon coupling, the dynamics of the organic cations, and the degree of lattice distortion. We report temperature-dependent Raman studies of BA_2_MAPb_2_I_7_ and BA_2_MA_2_Pb_3_I_10_ (BA = butylammonium; MA = methylammonium), which undergo two structural phase transitions. Raman data obtained in broad temperature (360–80 K) and wavenumber (1800–10 cm^−1^) ranges show that ordering of BA^+^ cations triggers the higher temperature phase transition, whereas freezing of MA^+^ dynamics occurs below 200 K, leading to the onset of the low-temperature phase transition. This ordering is associated with significant deformation of the inorganic sublattice, as evidenced by changes observed in the lattice mode region. Our results show, therefore, that Raman spectroscopy is a very valuable tool for monitoring the separate dynamics of different organic cations in perovskites, comprising “perovskitizer” and interlayer cations.

## 1. Introduction

Hybrid organic–inorganic lead halide perovskites have been widely studied in recent years due to their outstanding photovoltaic, light-emitting, nonlinear optical (NLO), and ferroelectric properties. These compounds may crystallize in various structures that exhibit significantly different properties. The three-dimensional (3D) perovskites of the general formula APbX_3_ (A = small organic cation, X = halide anion) are famous for their photovoltaic properties, which make them attractive materials for new-generation solar cells [1,2]. Unfortunately, the 3D perovskites are rare and can be formed for a few small organic cations only, such as methylammonium (MA^+^), formamidinium (FA^+^), aziridinium (AZR^+^), and methylhydrazinium (MHy^+^) [3,4,5,6,7,8,9]. One of the methods to enhance the diversity and modify the properties of these 3D lead halide perovskites is the preparation of mixed systems [10,11]. A more efficient way is, however, the employment of larger organic cations, which allow the synthesis of various lower-dimensional structures [12,13,14]. The most interesting ones are two-dimensional (2D) single-layered A′PbX_4_ or A″_2_PbX_4_ analogues (A′ and A″ are divalent and monovalent cations, respectively), composed of layers built up from corner-shared PbX_6_ octahedra separated by organic layers [14,15,16,17,18]. Such systems are natural quantum well structures, with much higher exciton binding energy and a blue shift of the bandgap compared to 3D analogues [14,15,16]. As a result, the photovoltaic performance of such 2D perovskites is poor compared to 3D analogues [19]. However, they are attractive materials for light-emitting, ferroelectric, and NLO applications [15,16,17,18,20]. To overcome problems with the large exciton binding energy of single-layered 2D perovskites, quasi-layered perovskites comprising two different cations can be synthesized. Most famous are Dion–Jacobson (DJ) (A′A_n−1_Pb_n_X_3n+1_) and Ruddlesden–Popper (RP) (A″_2_A_n−1_Pb_n_X_3n+1_) compounds (n indicates the number of octahedral layers within each inorganic slab), in which small cage cations A are located in the voids of inorganic slabs and large divalent (A′) or monovalent (A″) cations separate the slabs [11,18,21]. It is worth adding that in such systems, an increase in the inorganic slab thickness (number of layers, n) improves photovoltaic properties [21,22].

Lead halide perovskites are soft semiconductors in which optoelectronic properties, especially exciton binding energy and charge carrier transport, are strongly affected by changes in the strength of electron–phonon coupling [23,24,25]. This strength depends on the energy of phonon modes, the deformation of inorganic slabs, and the dynamics of organic cations [23,24,25]. The presence of organic components and the freezing of their dynamic molecular motions also afford an effective way for the generation of polar (ferro- or antiferroelectric) order [11,15,20,26,27]. It is therefore important to understand the effect of temperature on phonon properties, the dynamics of organic cations, and structural changes in hybrid perovskites. The most widely used method for such studies is Raman spectroscopy. This method has been employed for studies of many 3D and single-layer 2D lead halide perovskites [28,29,30,31,32,33,34,35,36]. Surprisingly, Raman studies of multilayered perovskites are very scarce. Fu et al. probed the low-wavenumber (200–20 cm^−1^) Raman modes of HA_2_GAPb_2_I_7_ and HA_2_MAPb_2_I_7_ (HA = hexylammonium) in the 300–77 K range, which revealed no phase transitions [37]. The low-wavenumber ranges of HA_2_GAPb_2_I_7_ (below 250 cm^−1^) and BA_2_MAPb_2_Br_7_ (150–10 cm^−1^) were also studied as a function of pressure to monitor distortion of the inorganic layers on compression [38,39]. RT Raman spectra below 200 cm^−1^ were presented for BA_2_APb_2_I_7_ (A = MA, FA, GA, dimethylammonium) and in the 1700–800 cm^−1^ range for BA_2_MA_2_Pb_3_I_10_ and BA_2_EA_2_Pb_3_I_10_ (EA = ethylammonium) [40,41]. Dahod et al. reported Raman spectra at RT and 77 K in the 150–10 cm^−1^ range for a range of RP phases (n = 2–4) comprising MA^+^ and FA^+^ cage cations and BA^+^ interlayer cations [42]. Recently, pressure-dependent Raman studies were reported for BA_2_MAPb_2_I_7_ in the 890–450 cm^−1^ range to monitor pressure-driven isomerism of BA^+^ cations [43]. This short overview of literature data shows that reports on Raman spectra of multi-layered perovskites were limited to narrow wavenumbers or temperature ranges.

Multi-layered RP iodides comprising BA^+^ and MA^+^ cations (n = 2–4) have been discovered by Stoumpos et al., and their crystal structures were reported as non-centrosymmetric at RT, i.e., *Cc*2*m* for BA_2_MAPb_2_I_7_ and *C*2*cb* for BA_2_MA_2_Pb_3_I_10_ [44]. This report suggests that these perovskites are promising candidates for solar cell and light-emitting applications. Later studies confirmed the good photovoltaic properties of these compounds [45,46] and showed that they also exhibit efficient NLO properties such as third harmonic generation (THG) and two-photon absorption [47]. Differential scanning colorimetry (DSC) showed that BA_2_MAPb_2_I_7_ (BA_2_MA_2_Pb_3_I_10_) exhibits two structural phase transitions observed on heating near 200 and 280 K (190 and 280 K) [48]. However, the nature of these phase transitions has not been studied. Cortecchia et al. confirmed that on cooling, BA_2_MAPb_2_I_7_ exhibits two structural phase transitions at 280.5 and 195.5 K associated with *Cc*2*m* → *P*$\bar{1}$ →*P*$\bar{1}$ symmetry change [49]. The higher (lower) temperature phase transition involved large (negligible) out-of-plane tilting of the octahedral units [49]. This paper did not provide any information on the contribution of BA^+^ and MA^+^ cation dynamics to the phase transition mechanism since BA^+^ and MA^+^ cations were assumed to be ordered in all phases. Two phase transitions were also reported for BA_2_MA_2_Pb_3_I_10_ at ~284 and ~185–190 K, but the crystal structures of the high-temperature (HT) and intermediate phases were solved as centrosymmetric, i.e., *Cmca* and *P*$\bar{1}$, respectively [50]. The centrosymmetric structure of the room-temperature phase, space group *Cmcm*, was also proposed for BA_2_MAPb_2_I_7_ [50]. Later studies revealed that BA_2_MAPb_2_I_7_ and BA_2_MA_2_Pb_3_I_10_ undergo phase transitions at 283.5 K (280 K) and 282 K (279 K) on heating and cooling, respectively, and that the phase stable below 280 K shows the presence of two different BA^+^ conformers [51]. This interesting report also showed that the phase transition is associated with a large change in the butyl CH_3_ group dynamics and that the dynamics of MA^+^ are similar to those in 3D MAPbI_3_ [51]. Very recently, quasi-elastic neutron scattering studies of BA_2_MAPb_2_I_7_ and BA_2_MA_2_Pb_3_I_10_ revealed that rotational freedom of the butyl CH_3_ group and MA^+^ cation freezes below 280 and 180 K, respectively [52]. It was also concluded that the rotational freedom of both cations is more restricted in the n = 2 compound [52].

Herein, we report temperature-dependent Raman studies of BA_2_MAPb_2_I_7_ and BA_2_MA_2_Pb_3_I_10_ to understand the effect of the inorganic slab thickness on the phonon properties of these perovskites. Our aim is also to understand the mechanism of the phase transitions and temperature-induced changes in the lattice dynamics and how the behavior of multi-layered perovskites differs from the behavior of 3D MAPbI_3_.

## 2. Materials and Methods

### 2.1. Materials and Synthesis

Single crystals of BA_2_MAPb_2_I_7_ and BA_2_MA_2_Pb_3_I_10_ were grown in the same method as recently reported for MA_1-x_A_x_PbI_3_ systems (A = EA, MHy) [53,54]. In this method, stoichiometric amounts of PbI_2_, n-butylamine (99.5%, Sigma-Aldrich, St. Louis, MO, USA), and methylamine (2M solution in methanol, Sigma-Aldrich, St. Louis, MO, USA) were dissolved in a mixture of propylene carbonate (99.7%, Sigma-Aldrich, St. Louis, MO, USA) and hydroiodic acid (57 wt% in H_2_O, Sigma-Aldrich, St. Louis, MO, USA). The HI/propylene carbonate ratio was 1:2.7, and the total amount of reagents corresponded to one mmol of the target compounds. The clear solutions were transferred to glass vials, which were closed and kept at 50 °C. Dark purple (BA_2_MA_2_Pb_3_I_10_) and purple (BA_2_MAPb_2_I_7_) plate-like crystals were separated from the liquid and dried at RT. A good agreement of their powder diffraction patterns with the simulated ones based on single-crystal data reported in [44] confirmed the phase purity of the obtained bulk samples (Figure S1).

### 2.2. Raman Spectroscopy

Temperature-dependent Raman spectra of BA_2_MAPb_2_I_7_ and BA_2_MA_2_Pb_3_I_10_ crystals in the 1700–100 cm^−1^ range were measured using a Renishaw inVia Raman spectrometer (Renishaw, Wotton-under-Edge, UK), equipped with a confocal DM2500 Leica optical microscope (Renishaw, Wotton-under-Edge, UK), a thermoelectrically cooled CCD as a detector (Renishaw, Wotton-under-Edge, UK), and a diode laser operating at 830 nm (Renishaw, Wotton-under-Edge, UK). A 20× microscope magnification lens was used; the size of the studied crystals was less than 0.5 mm, and the laser spot diameter was about 0.75 μm. We could not record the spectra in the N-H and C-H stretching regions (above 2800 cm^−1^) since the higher wavenumber range of the used CCD detector combined with the 830 nm laser is 1800 cm^−1^. The same spectrometer was used to record Raman spectra in the low-wavenumber range (200–10 cm^−1^), but in this case an eclipse filter (Renishaw, Wotton-under-Edge, UK) was employed. This range was measured since it provides information on the lattice modes and, thus, the long-range order and distortion of the inorganic sublattice. The temperature was controlled using a THMS600 stage (Linkam, Tadworth, UK), and the spectral resolution was 2 cm^−1^. The temperature ranges, 360–80 and 320–80 K for BA_2_MAPb_2_I_7_ and BA_2_MA_2_Pb_3_I_10_, respectively, were chosen to cover the temperatures at which phase transformations occur. The lowest temperature was 80 K due to the limitations of the Linkam stage.

## 3. Results and Discussion

### 3.1. Temperature-Dependent Raman Study of BA_2_MAPb_2_I_7_

**Figure 1:**
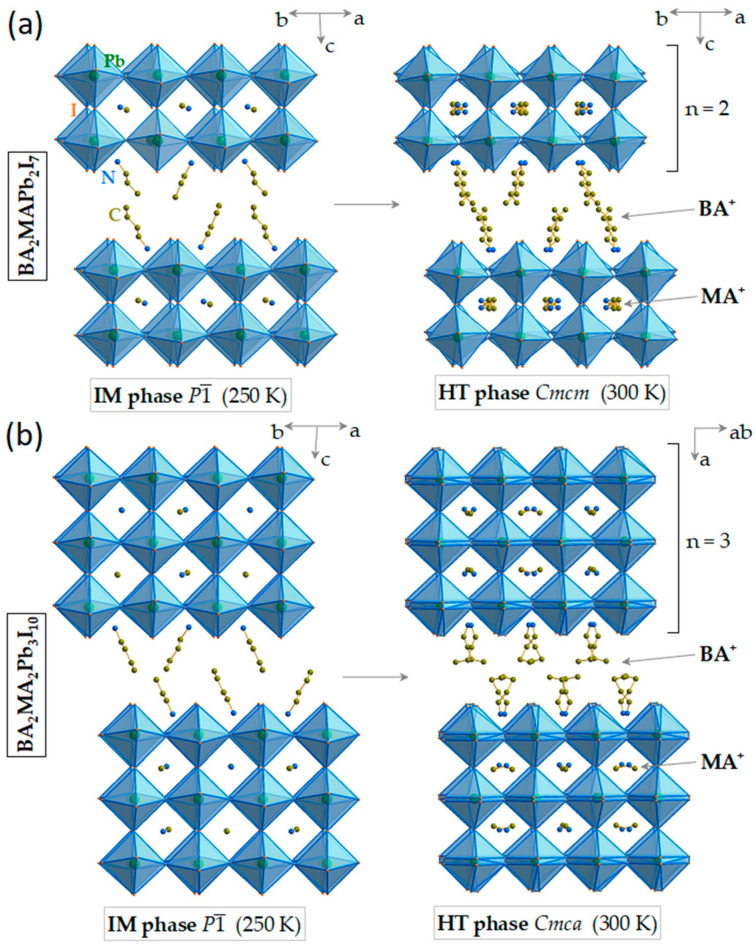
Crystal structures of the high-temperature (HT) and intermediate (IM) phases of (**a**) BA_2_MAPb_2_I_7_ and (**b**) BA_2_MA_2_Pb_3_I_10_.

Room-temperature and intermediate structures of BA_2_MAPb_2_I_7_ are presented in Figure 1a. These structures consist of corner-shared PbI_6_ octahedra forming inorganic slabs composed of two perovskite layers. MA^+^ cations are located inside the perovskite voids, while BA^+^ cations separate the inorganic slabs. Both structures differ in respect to organic cation disorder and tilts of PbI_6_ octahedra, as mentioned in the Section 1. Further crystallographic details can be found in [50].

**Figure 2:**
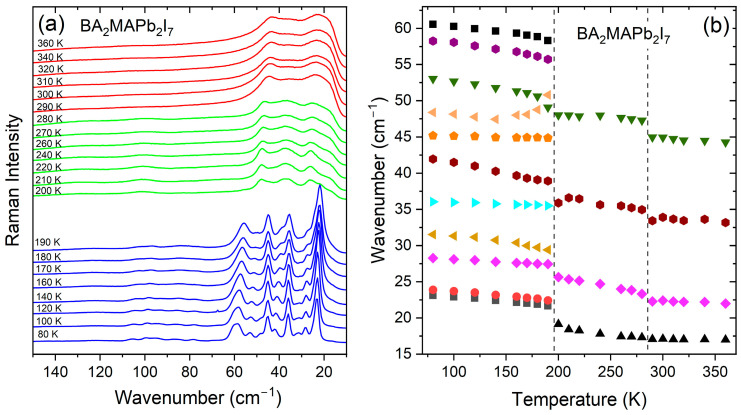
Temperature-dependent Raman spectra in the 150–10 cm^−1^ range (**a**) and plots of Raman wavenumbers for lattice modes (**b**) of BA_2_MAPb_2_I_7_. Red, green, and blue colors correspond to the HT, intermediate, and LT phases, respectively. Vertical lines indicate phase transition temperatures.

**Figure 3:**
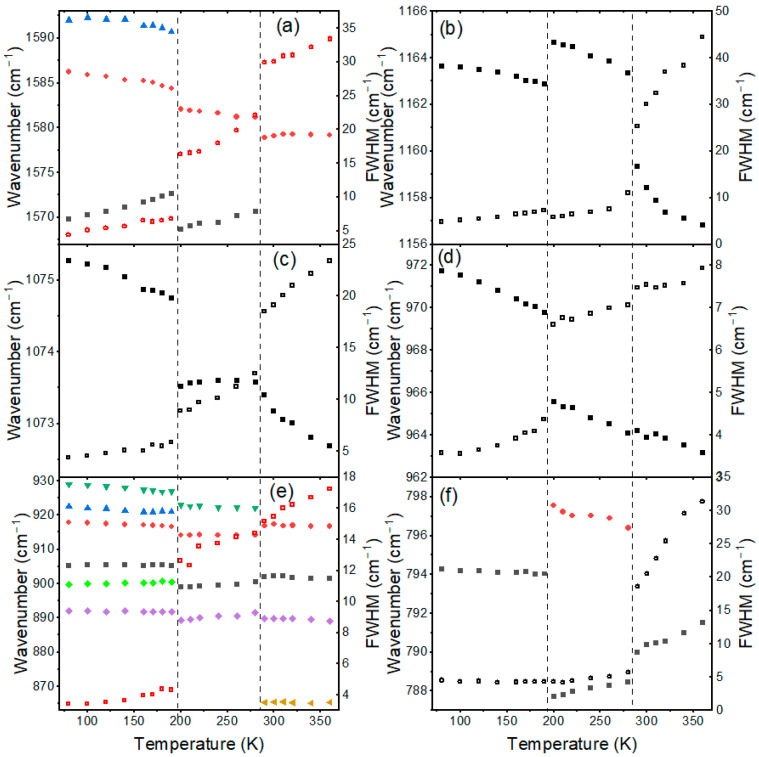
Plots of Raman wavenumbers (closed symbols) and FWHM (open symbols) for selected internal modes of BA_2_MAPb_2_I_7_: (**a**) δ_as_(NH_3_^+^), (**d**) ν(CN) and (**e**) ρ(NH_3_^+^) + ρ(CH_3_) modes of MA^+^ as well as (**a**) δ_as_(NH_3_^+^), (**b**) ρ(NH_3_^+^), (**c**) ν_as_(CC), (**e**) ω(NH,CH), ν_s_(CC), ν_s_(CN) and (**f**) ρ(CH_2_) modes of BA^+^. The same color denotes FWHM and wavenumber data for the same mode. Vertical lines denote phase transition temperatures.

The temperature-dependent Raman spectra of a BA_2_MAPb_2_I_7_ single crystal are presented in Figure 2a and Figure S2, whereas plots of the wavenumbers and full width at half-maximum (FWHM) values vs. temperature are presented in Figure 2b and Figure 3. The observed modes are listed in Table S1 together with the assignment based on previous Raman scattering studies of MA-based perovskites [29,32,33,34] and compounds comprising BA^+^ cations [41,55,56]. All modes observed above 300 cm^−1^ can be attributed to internal vibrations of MA^+^ and BA^+^ cations (Table S1). The broad band at 238 cm^−1^ (value at 280 K) can be attributed to the MA-cage mode, i.e., the mode that involves the C–N torsion [29,34]. Interestingly, this mode was observed at a very similar wavenumber in MAPbI_3_ (241 cm^−1^ at RT [34]). This behavior shows that the separation of double octahedral layers by interlayer BA^+^ cations weakly affects interactions between MA^+^ cations and PbI_6_ octahedra. In the lattice modes region, we attribute the bands below 32 cm^−1^ to octahedra librations (or alternatively to octahedra twist), those in the 68–33 cm^−1^ range to Pb-I bending (or alternatively to octahedra distortion), and the remaining bands, observed in the 200–70 cm^−1^ range, to coupled modes involving librations and translations of MA^+^ and BA^+^ as well as Pb–I stretching modes (Table S1).

Raman spectra of the high-temperature (HT) phase in the lattice modes region are dominated by very strong and broad bands observed below 44 cm^−1^ (Figure 2a, Table S1). Other lattice modes are visible at 132 and 104 cm^−1^ as shoulders (Table S1). The very large width of the lattice bands points to a pronounced disorder of the HT phase. When the temperature decreases, clear changes can be observed at 280 K. Firstly, Raman bands exhibit weak shifts (Figure 2a and Figure 3). Secondly, the relative intensity of the Raman bands changes (Figure 2a). Thirdly, Raman bands narrow, but this narrowing is rather weak, and the number of observed Raman bands does not change (Figure 2). The observed changes in the Raman spectra indicate that the phase transition from the HT phase to the intermediate phase weakly affects the inorganic slabs, suggesting that the major contribution to the PT mechanism comes from the interlayer BA^+^ cations. On further cooling, abrupt narrowing and splitting of the Raman bands occur at 190 K (Figure 2). It can also be noticed that the bands exhibit a significant shift to higher wavenumbers (Figure 2b and Table S1). Raman spectra show, therefore, that the PT from the intermediate phase to the low-temperature (LT) phase near 190 K leads to abrupt freezing of molecular motions, strong distortion of the inorganic slabs, and hardening of the lattice.

To understand the impact of organic cation dynamics on the mechanism of phase transitions, we have also studied the temperature dependence of the internal modes. Figure S2 and Figure 3 show that many bands related to BA^+^ and MA^+^ cations are very broad in the HT phase, confirming the disorder of organic cations. Upon cooling, sudden changes can be observed when the temperature decreases from 290 to 280 K (Figure S2 and Figure 3). Firstly, some bands related to BA^+^ cations exhibit sudden narrowing. For instance, FWHM of the ρ(NH_3_^+^), ν_as_(CC), and ρ(CH_2_) modes of BA^+^ decreases from 25.2, 18.5, and 18.6 cm^−1^ at 290 K to 11.0, 12.5, and 5.7 cm^−1^ at 280 K (Figure 3b,c,f). On the other hand, the MA-related bands do not show significant narrowing at the phase transition temperature (for instance, the ν(CN) and ρ(NH_3_^+^) + ρ(CH_3_) modes in Figure 3d,e; note that the apparent narrowing for the δ_as_(NH_3_^+^) mode of MA^+^ seen in Figure 3a is largely due to the fact that above 290 K the band was fitted as a singlet and at 280 K as a doublet). Secondly, Raman bands exhibit weak shifts, up to 4 cm^−1^ for the ρ(NH_3_^+^) mode of BA^+^ (Figure 3 and Figure S2, Table S1). Temperature-dependent spectra of internal modes indicate, therefore, that this phase transition is triggered by the ordering of BA^+^ cations while the dynamics of MA^+^ cations are weakly affected.

Abrupt changes can also be noticed when the temperature decreases from 200 to 190 K (Figure 3 and Figure S2). First of all, sudden narrowing is observed for the MA-related bands. For instance, FWHM of the δ_as_(NH_3_^+^), ν(CN), and ρ(NH_3_^+^) + ρ(CH_3_) modes of MA^+^ decreases from 16.3, 6.6, and 12.6 cm^−1^ at 200 K to 6.8, 4.3, and 4.3 cm^−1^ at 190 K (Figure 3a,d,e). Bands related to BA^+^ vibrations exhibit either negligible (for instance, the ρ(NH_3_^+^) and ρ(CH_2_) modes in Figure 3b,f) or much less pronounced changes in FWHM (see the ν_as_(CC) band in Figure 3c, which narrows from 8.9 cm^−1^ at 200 K to 5.9 cm^−1^ at 190 K). Secondly, Raman bands exhibit significant shifts, usually to higher wavenumbers. This behavior is observed for both cations (Figure 3 and Figure S2, Table S1). Thirdly, many bands split into doublets (Table S1). Fourthly, Raman bands exhibit sudden changes in relative intensity (Figure S2). Changes in the Raman spectra prove that the phase transition from the intermediate phase to the LT phase is triggered by the ordering of MA^+^ cations, which increases hydrogen bond strength and shortens the C-N bond of MA^+^. The dynamics of BA^+^ cations do not contribute significantly to the phase transition mechanism, but distortion induced in the inorganic slabs also affects BA-framework interactions, as evidenced by significant shifts in BA-related modes. Since these modes exhibit shifts to higher values, Raman data suggest that the phase transition leads to strengthening of the hydrogen bonds formed between BA^+^ and Br^−^.

It is important to compare our results with the previous crystallographic, solid-state NMR, and quasi-elastic neutron scattering data [49,51,52]. Raman data confirm conclusions derived from NMR and neutron scattering studies that the phase transition near 280 K is related to the freezing of the butyl CH_3_ group dynamics, while that near 190 K is due to the freezing of the rotational freedom of the MA^+^ cation [51,52]. Crystallographic and NMR data also suggested that the phases stable below 290 K comprise two different BA^+^ conformers. Raman data show the presence of two unique BA^+^ cations through the splitting of many BA-related bands into doublets in the LT phase but no splitting of BA^+^ bands in the intermediate phase. It is likely that the expected splitting is not observed in the intermediate phase since, due to its weak magnitude and broadening of bands, separate bands could not be resolved. Crystallographic data also revealed that the phase transition near 290 K leads to a decrease in symmetry from orthorhombic to triclinic and that it is triggered by tilts of BA^+^ cations and PbI_6_ octahedra without any pronounced distortion of the octahedra [49]. Interestingly, in spite of strong symmetry change and pronounced out-of-plane tilts of PbI_6_ octahedra, Raman spectra in the lattice modes region show weak changes and no splitting. We argue that this effect is related to both the large width of the lattice bands, the weak sensitivity of these modes to out-of-plane tilts, and the small distortion of the PbI_6_ units in the intermediate phase. According to the structural data, the LT phase is also triclinic, and the phase transition near 190 K leads to a pronounced increase in the octahedral distortion [49]. Our Raman spectra show drastic changes at this phase transition in the lattice modes region, i.e., large shifts and splitting of modes, which resembles changes observed in 3D MAPbBr_3_ and MAPbI_3_ at the tetragonal-to-orthorhombic phase transition at 148 K associated with freezing of MA^+^ motions [29,33]. This behavior points to very similar mechanisms for these LT-phase transitions in BA_2_MAPb_2_I_7_ and 3D analogues. We suppose that such pronounced sensitivity of Raman spectra to this phase transition, in spite of the same triclinic symmetry of both LT and intermediate phases of BA_2_MAPb_2_I_7_, can be attributed to the fact that this phase transition leads to strong distortion of PbI_6_ units and narrowing of bands due to locking of MA^+^ dynamics, which facilitate observation of the phase transition-induced changes in the lattice mode region. Thus, our Raman data indicate that distortion of the lead– halide octahedra has a much more pronounced effect on the lattice modes than their out-of-plane tilts or symmetry changes.

The presence of structural phase transitions should affect the optoelectronic application of BA_2_MAPb_2_I_7_ because they affect octahedral tilts, distortion of the framework, dielectric screening of free carriers and phonon energies, and thus electron–phonon coupling and exciton binding energy. In this respect, literature data on lead halide perovskites showed that changes in Pb-X bond lengths, tilts, and distortions of PbX_6_ octahedra affect the band gap and PL [5,49,50]. First of all, an increase in the octahedral tilting (decrease of the Pb-(µ-I)-Pb angles from the ideal 180°) leads to weaker interaction between Pb s orbitals and I p_x_ and p_y_ orbitals in the valence band maximum, mixing of I p and s orbitals, as well as an increase in the antibonding interaction in the conduction band maximum [50]. These effects lead to a blue shift in the band gap. On the other hand, a decrease in the equatorial Pb-I bond lengths leads to an increase in the antibonding interaction at the valence bond maximum [50]. As a result, the band gap narrows with decreasing Pb-I bond lengths. Former PL studies [50] are consistent with our Raman data and previous structural data [49] since they revealed a weak blue shift of a few nm at the HT-intermediate phase transition, where Raman data show weak changes in the lattice modes region due to weak distortion of the PbI_6_ octahedra, and a strong blue shift exceeding 20 nm at the intermediate-LT phase transition, where Raman data show drastic changes in the lattice modes region due to very large octahedral distortion. It is worth noting that in the case of 3D MAPbI_3_, the tetragonal to orthorhombic phase transition associated with the freezing of MA^+^ dynamics and strong distortion of the inorganic subnetwork also led to a very large blue shift of about 20 nm [57] and drastic changes in the Raman spectra in the lattice modes region [29]. This result confirms the same mechanism of the LT phase transitions in 3D and 2D analogues and a similar distortion of the inorganic subnetwork. It also shows that there is a clear correlation between changes in the lattice modes at the phase transition and the blue shift of the photoluminescence.

Regarding implications of the observed structural changes and lattice dynamics on photovoltaic properties, it is important to note that according to former studies of MAPbX_3_ (X = Br, I) perovskites, mobility and lifetime of charge carriers increase when the temperature decreases due to a slowing down of molecular dynamics. An especially pronounced increase by a factor of 3–6 was observed at the LT phase transition when the reorientational motions of MA^+^ cations were locked [58]. Studies of the photovoltaic behavior of MAPbI_3_ showed, however, that whereas the power conversion efficiency (PCE) was weakly affected by the cubic-tetragonal phase transition, it strongly decreased in the LT orthorhombic phase [59]. Our Raman data show that the HT-intermediate (intermediate-LT) phase transition has a weak (strong) effect on the octahedral distortion and dynamics of MA^+^ cations. Therefore, Ramana data show that the HT-intermediate (intermediate-LT) phase transition should weakly (strongly) affect the photovoltaic performance.

### 3.2. Temperature-Dependent Raman Study of BA_2_MA_2_Pb_3_I_10_

Figure 1b shows that, similarly to BA_2_MAPb_2_I_7_, the crystal structures of BA_2_MA_2_Pb_3_I_10_ also consist of inorganic slabs separated by BA^+^ cations. However, in this case, the inorganic slabs are composed of three inorganic layers.

**Figure 4:**
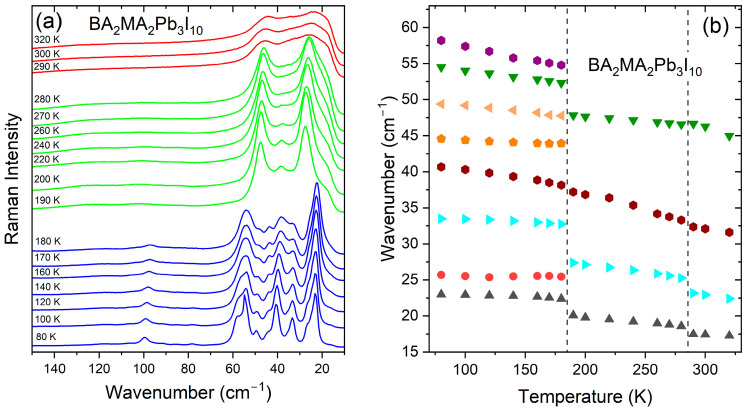
Temperature-dependent Raman spectra in the 150–10 cm^−1^ range (**a**) and plots of Raman wavenumbers for lattice modes (**b**) of BA_2_MA_2_Pb_3_I_10_. Red, green, and blue colors correspond to the HT, intermediate, and LT phases, respectively. Vertical lines denote phase transition temperatures.

**Figure 5:**
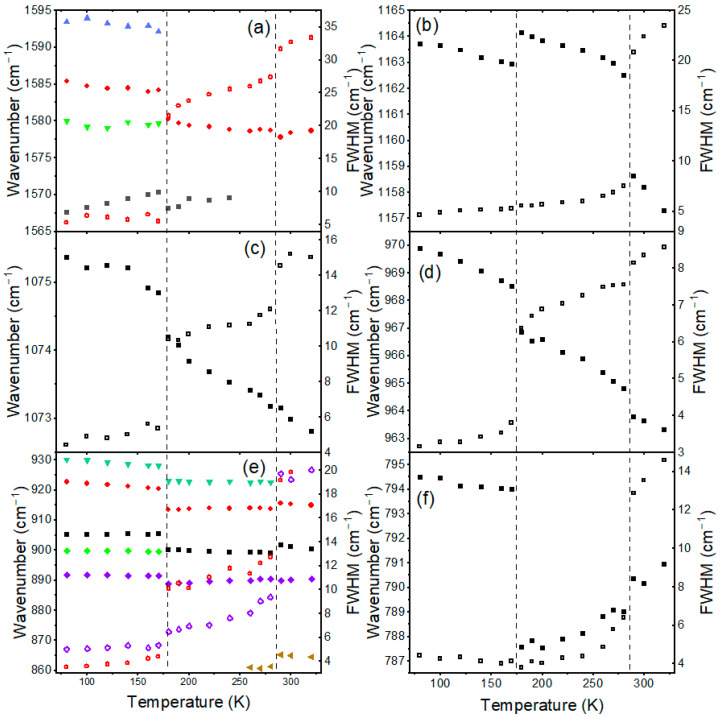
Plots of Raman wavenumbers (closed symbols) and FWHM (open symbols) for selected internal modes of BA_2_MA_2_Pb_3_I_10_: (**a**) δ_as_(NH_3_^+^), (**d**) ν(CN), and (**e**) ρ(NH_3_^+^) + ρ(CH_3_) modes of MA^+^ as well as (**a**) δ_as_(NH_3_^+^), (**b**) ρ(NH_3_^+^), (**c**) ν_as_(CC), (**e**) ω(NH,CH), ν_s_(CC), ν_s_(CN), and (**f**) ρ(CH_2_) modes of BA^+^. The same color denotes the FWHM and wavenumber data for the same mode.

The temperature-dependent Raman spectra of a BA_2_MA_2_Pb_3_I_10_ single crystal are presented in Figure 4a and Figure S3, whereas plots of the wavenumbers and FWHM values vs. temperature are presented in Figure 4b and Figure 5. The observed modes are listed in Table S2. The Raman spectra of the HT phase of BA_2_MA_2_Pb_3_I_10_ are very similar to the spectra of BA_2_MAPb_2_I_7_. In particular, in the internal mode’s region, no clear shifts of bands can be observed at 290 K between the two compounds (Figures S2–S4). Closer inspection shows, however, that the lattice modes of BA_2_MA_2_Pb_3_I_10_ shift slightly (by less than 2 cm^−1^) to higher wavenumbers, and the Raman bands in the lattice modes region are broader compared to BA_2_MAPb_2_I_7_ (Figure 2, Figure 4 and Figure S5). These changes suggest larger disorder and shorter Pb-I bonds in the HT phase of tri-layered analogue, probably due to a larger octahedral distortion.

Changes in the Raman spectra of BA_2_MA_2_Pb_3_I_10_ due to the onset of the structural phase transitions are very similar to those discussed above for BA_2_MAPb_2_I_7_, i.e., the HT (LT) phase transition leads to weak (strong) narrowing of the lattice modes, strong (weak) narrowing of BA-related bands, and weak (strong) narrowing of MA-related bands (Figure 4a and Figures S3–S5). Furthermore, the LT phase transition leads to the splitting of lattice modes and many internal modes into two or three components (Figure 4, Figure 5 and Figures S3–S5, Table S2). Many internal modes also exhibit significant shifts, especially at the LT phase transition (Figure 5 and Figure S3). Raman spectra indicate, therefore, that BA_2_MA_2_Pb_3_I_10_ exhibits very similar structural changes as its two-layered analogue, i.e., the HT (LT) phase transition is triggered by the freezing of BA^+^ (MA^+^) dynamics, which leads to weak (or strong) distortion of the octahedral slabs.

Let us now discuss some differences in the evolution of Raman spectra at phase transition temperatures. In the lattice modes region, the main difference in the spectra of the intermediate phases (at 280 K) can be seen in the increased or decreased intensity of the ~47 and ~23 cm^−1^ (~32 cm^−1^) bands of BA_2_MA_2_Pb_3_I_10_ (Figure 4 and Figure S5). Much larger differences are seen, however, when the compounds transform into the LT phases. In particular, many lattice modes of BA_2_MA_2_Pb_3_I_10_ are observed at lower wavenumbers compared to BA_2_MAPb_2_I_7_ (Figure 2, Figure 4 and Figure S5; Tables S1 and S2). This behavior suggests that the LT phase of tri-layered perovskite is less distorted than the LT phase of the two-layered analogue. In the internal modes region at 280 K, the most obvious difference is the larger wavenumber of the MA-cage mode of BA_2_MAPb_2_I_7_ (238 cm^−1^) than BA_2_MA_2_Pb_3_I_10_ (234 cm^−1^). Since the energy of this mode depends strongly on the size of the perovskite cage, as evidenced by the large shift of this mode to lower wavenumbers in 3D analogues when Cl^−^ is replaced by larger Br^−^ and then I^−^ [34], the shift of the MA-cage mode to lower wavenumbers for BA_2_MA_2_Pb_3_I_10_ indicates weakening of MA-cage interactions and thus longer MA^…^I distance when going from two-layered to three-layered analogue. The same effect is also observed in the LT phases, where the MA-cage mode is observed at 240 cm^−1^ for BA_2_MAPb_2_I_7_ and 237 cm^−1^ for BA_2_MA_2_Pb_3_I_10_ (Tables S1 and S2). Significant differences in the MA-framework interactions are also reflected in the behavior of other MA-related bands. For instance, the δ(CN), ν(CN), and ρ(NH_3_^+^) + ρ(CH_3_) modes observed at 1260, 972, and 918 cm^−1^ for BA_2_MAPb_2_I_7_ shift to 1255, 970, and 923 cm^−1^ for BA_2_MA_2_Pb_3_I_10_. Contrary to the MA-based modes, BA-related modes’ wavenumbers do not show any significant dependence on the thickness of octahedral slabs.

It is worth adding that the shifts and broadening of the MA-related bands of BA_2_MAPb_2_I_7_ and BA_2_MA_2_Pb_3_I_10_ are similar to those reported for MAPbI_3_ [29]. For instance, during the phase transition to the LT phase, FWHM (wavenumber) of the δ_as_(NH_3_^+^), ν(CN), and ρ(NH_3_^+^) + ρ(CH_3_) modes decreased (increased) by 9.5, 2.2, and 8.3 cm^−1^ (2.3, 4.2, and 2.5 cm^−1^) for BA_2_MAPb_2_I_7_, 16.0, 2.6, and 5.7 cm^−1^ (3.9, 1.7, and 6.8 cm^−1^) for BA_2_MA_2_Pb_3_I_10_, and about 28, 2, and 10 cm^−1^ (4, 2, and 5 cm^−1^) for MAPbI_3_ [29]. This result confirms that the dynamics of MA^+^ in the RP phases are similar to those in 3D MAPbI_3_ and that it freezes at low temperatures.

Although BA_2_MAPb_2_I_7_ and BA_2_MA_2_Pb_3_I_10_ show very similar temperature dependence in Raman spectra, charge carrier mobility near room temperature might be slightly smaller in the latter case due to more pronounced MA^+^ dynamics in BA_2_MA_2_Pb_3_I_10_ resulting from weaker MA-framework interactions. Weaker distortion of PbI_6_ octahedra at the intermediate-LT phase transition in BA_2_MA_2_Pb_3_I_10_ compared to BA_2_MAPb_2_I_7_ suggests that this transformation should have a weaker effect on photovoltaic and photoluminescence properties in the tri-layered analogue.

## 4. Conclusions

We have performed temperature-dependent Raman studies of two RP iodides comprising BA^+^ and MA^+^ cations. We proposed the assignment of Raman-active modes. The smaller width of Raman bands in the lattice region observed for the HT phase of BA_2_MAPb_2_I_7_ compared to BA_2_MA_2_Pb_3_I_10_ revealed that the lattice dynamics of organic cations are more restricted in the two-layered analogue. The increase in inorganic slab thickness has negligible effect on BA^+^ modes but affects MA-related modes, indicating that thickness affects hydrogen-bond interactions between MA^+^ and I^−^.

Temperature-dependent studies revealed that in both compounds, the HT-intermediate phase transition is triggered by changes in BA^+^ dynamics, probably by freezing of the BA-related CH_3_ group reorientational motions, and this change is associated with tilts of BA^+^ cations without any pronounced distortion of the inorganic slabs. The intermediate-LT phase transition is triggered by the freezing of MA^+^ reorientational motions, which leads to pronounced distortion of the inorganic slabs. This type of structural change is very similar to that observed in 3D MAPbI_3_, but separation of inorganic slabs by BA^+^ cations leads to a significant increase in the phase transition temperatures from ~160 K for MAPbI_3_ to ~185–190 K for BA_2_MA_2_Pb_3_I_10_ and 195.5 K for BA_2_MAPb_2_I_7_. This behavior indicates that restriction of the rotational freedom of MA^+^ increases with decreasing thickness of the inorganic slabs. We also show that there is a clear correlation between changes in the lattice modes at the phase transitions and blue shifts in the photoluminescence. Since multilayered systems pose a significant challenge to crystal structure solutions, Raman spectroscopy provides an alternative for monitoring the degree of octahedral distortion in order to understand temperature-dependent changes in band gaps and photoluminescence. In summary, we show that vibrational spectroscopy is a very valuable tool in studies of hybrid perovskites comprising two different organic cations since it allows to obtain information on the distortion of inorganic sublattice and to monitor molecular dynamics separately for each cation, thus helping to understand the mechanism of the structural phase transitions and the effect of structural changes on the optoelectronic properties of these compounds.

## Data Availability

The data presented in this study are available at: https://doi.org/10.5281/zenodo.11147550.

## Supplementary Materials

The following are available online at https://www.mdpi.com/article/10.3390/ma17112503/s1. Table S1: Raman wavenumbers for BA_2_MAPb_2_I_7_ at 360, 280, and 80 K. Table S2: Raman wavenumbers for BA_2_MA_2_Pb_3_I_10_ at 320, 280, and 80 K. Figure S1: Experimental and simulated powder X-ray diffraction patterns of BA_2_MAPb_2_I_7_ and BA_2_MA_2_Pb_3_I_10_. Figure S2: Temperature-dependent Raman spectra of BA_2_MAPb_2_I_7_ in the 1700–100 cm^−1^ range. Figure S3: Temperature-dependent Raman spectra of BA_2_MA_2_Pb_3_I_10_ in the 1640–200 cm^−1^ range. Figure S4: Comparison of Raman spectra of BA_2_MAPb_2_I_7_ and BA_2_MA_2_Pb_3_I_10_ corresponding to three phases at 290, 280, and 80 K in the internal modes region. Figure S5: Comparison of Raman spectra of BA_2_MAPb_2_I_7_ and BA_2_MA_2_Pb_3_I_10_ corresponding to three phases at 290, 280, and 80 K in the lattice modes region.
