# Supplementary material for: Phonon Properties and Lattice Dynamics of Two- and Tri-Layered Lead Iodide Perovskites Comprising Butylammonium and Methylammonium Cations—Temperature-Dependent Raman Studies
Source: Materials (Basel). 2024 May 22;17(11):2503. doi: 10.3390/ma17112503 (PMC11172726; doi:10.3390/ma17112503)
Supplement: Supplementary file 1 [file materials-17-02503-s001.zip › materials-3002260-supplementary.pdf]

Supporting information for

Phonon properties and lattice dynamics of two- and tri-layered lead iodide perovskites comprising butylammonium and methylammonium cations – temperature-dependent Raman studies

Mirosław Mączka , Szymon Smółka and Maciej Ptak*

Institute of Low Temperature and Structure Research, Polish Academy of Sciences, Okólna 2, 50-422 Wrocław , Poland

e-mail: m.maczka@intibs.pl

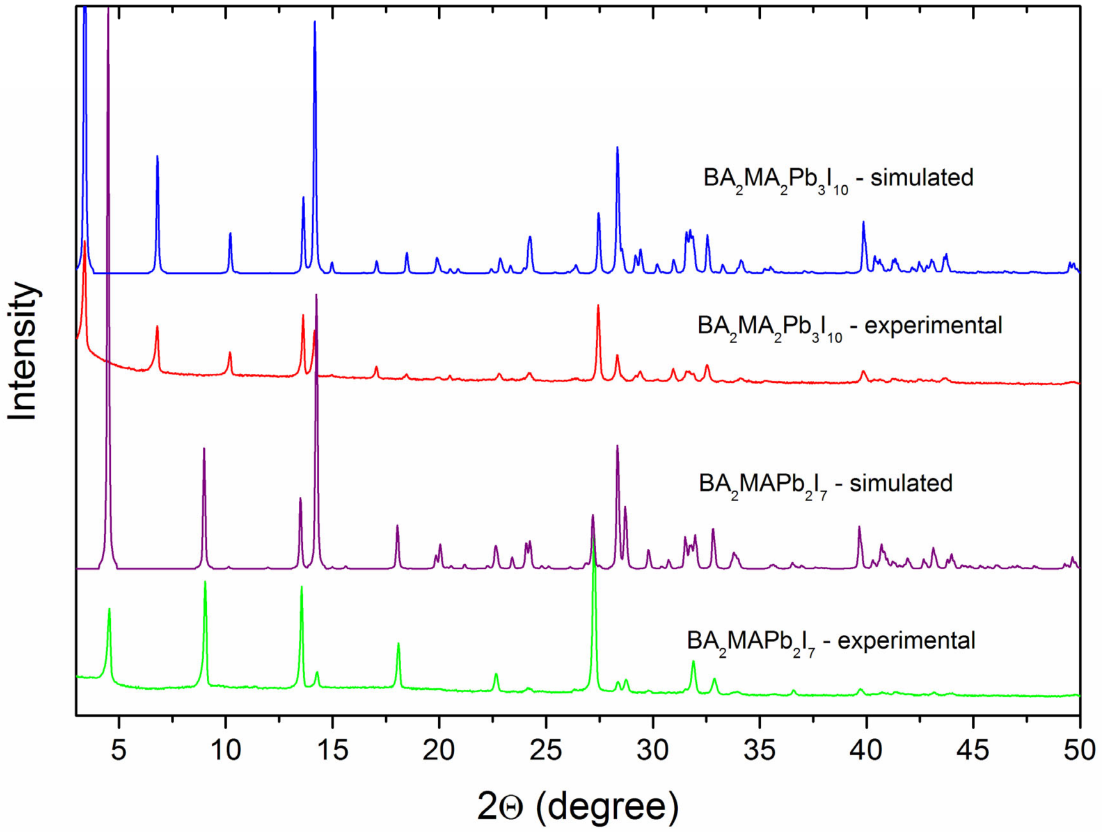

Figure S1. Experimental and simulated powder X-ray diffraction patterns of $BA_2MAPb_2I_7$ and $BA_2MA_2Pb_3I_{10}$.

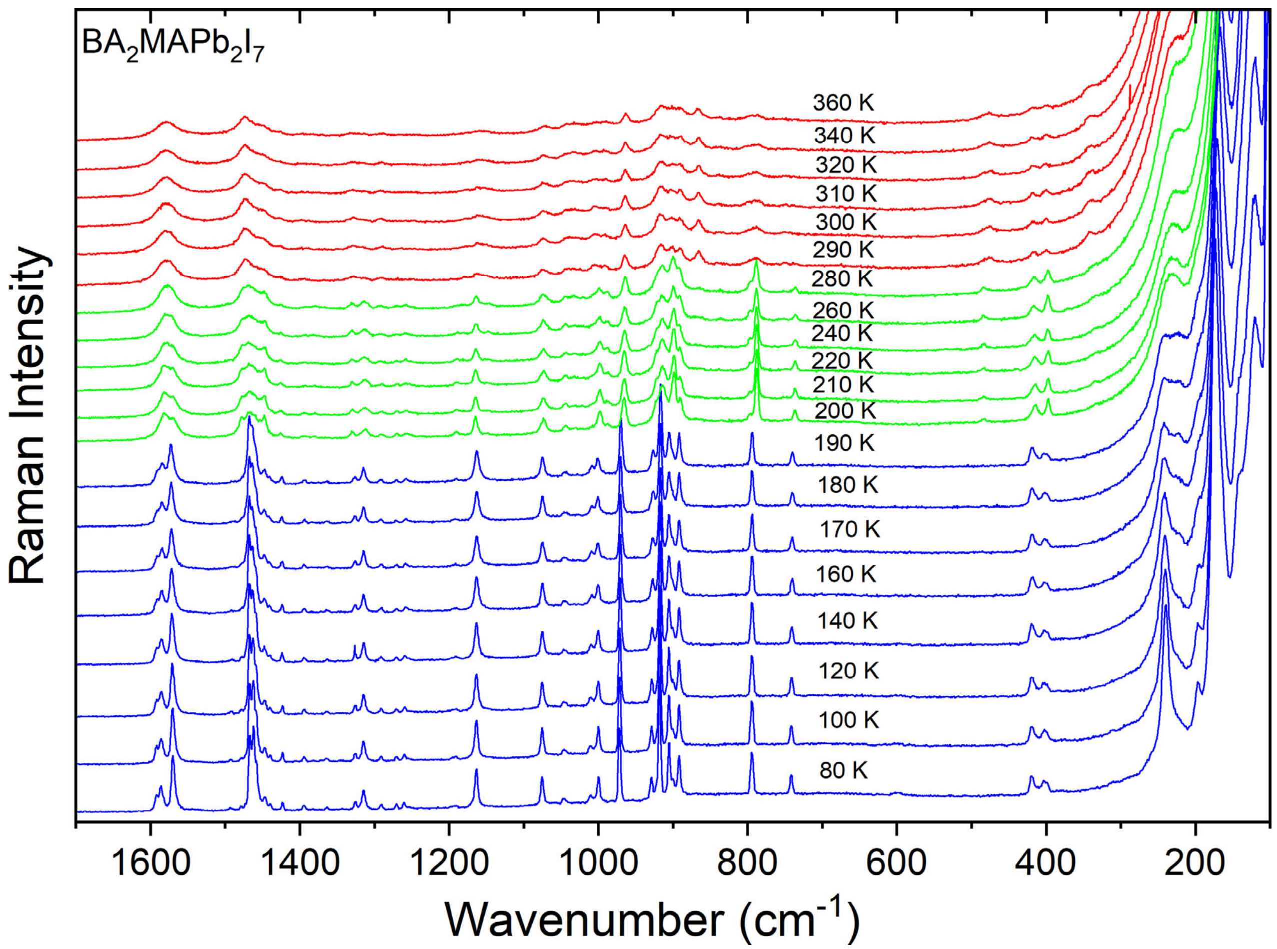

Figure S2. Temperature-dependent Raman spectra of $BA_2MAPb_2I_7$ in the 1700-100 cm^{-1} range.

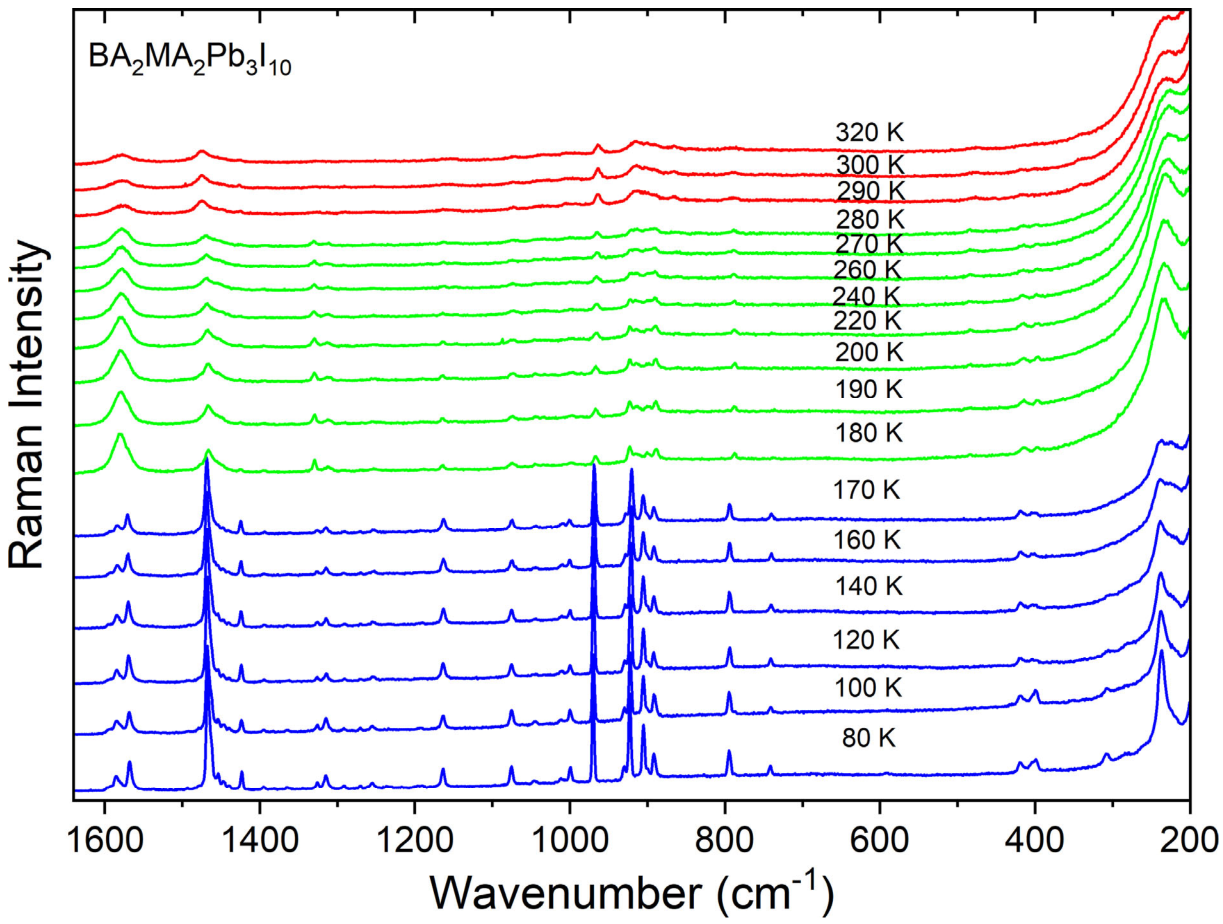

Figure S3. Temperature-dependent Raman spectra of $BA_2MA_2Pb_3I_{10}$ in the 1640-200 cm^{-1} range.

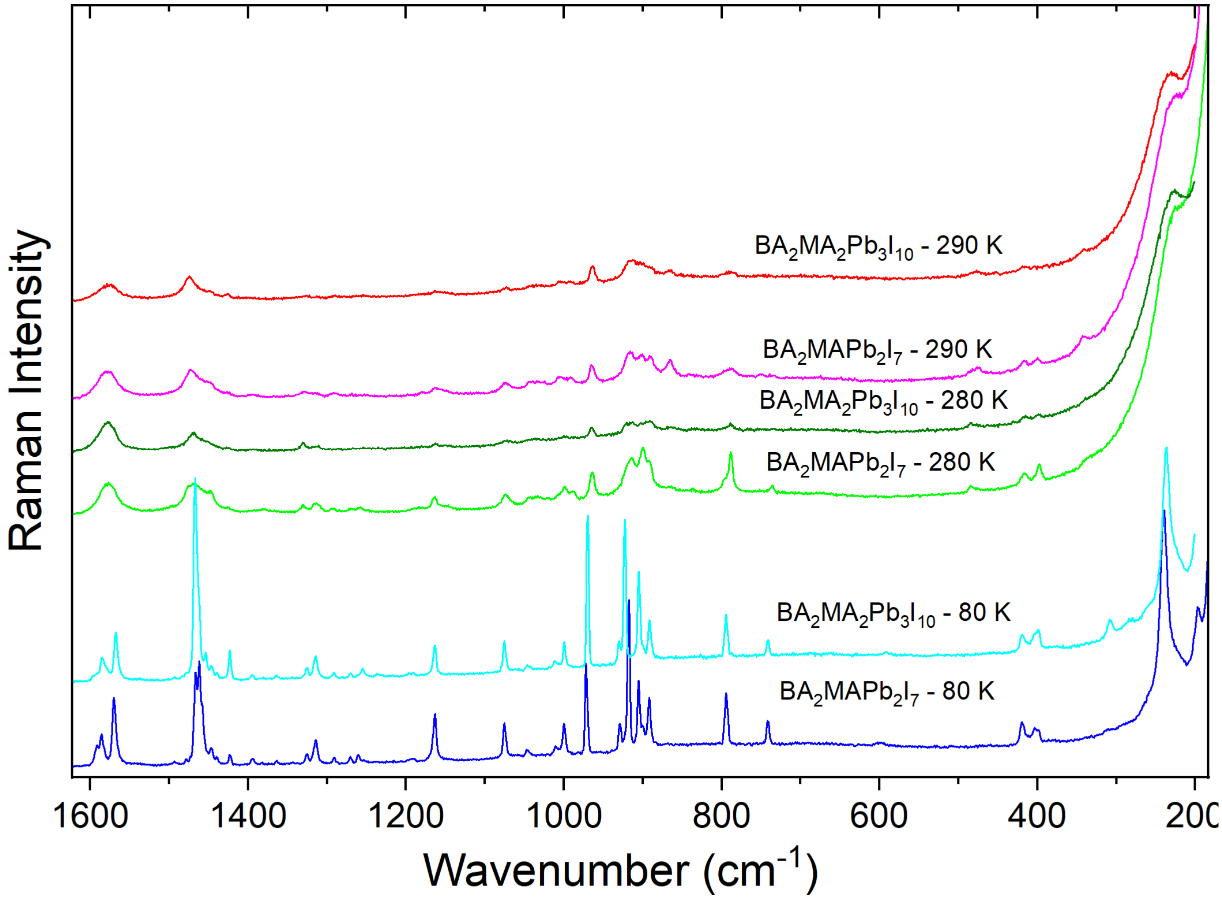

Figure S4. Comparison of Raman spectra of $BA_2MAPb_2I_7$ and $BA_2MA_2Pb_3I_{10}$ corresponding to three phases at 290, 280 and 80 K in the internal modes region.

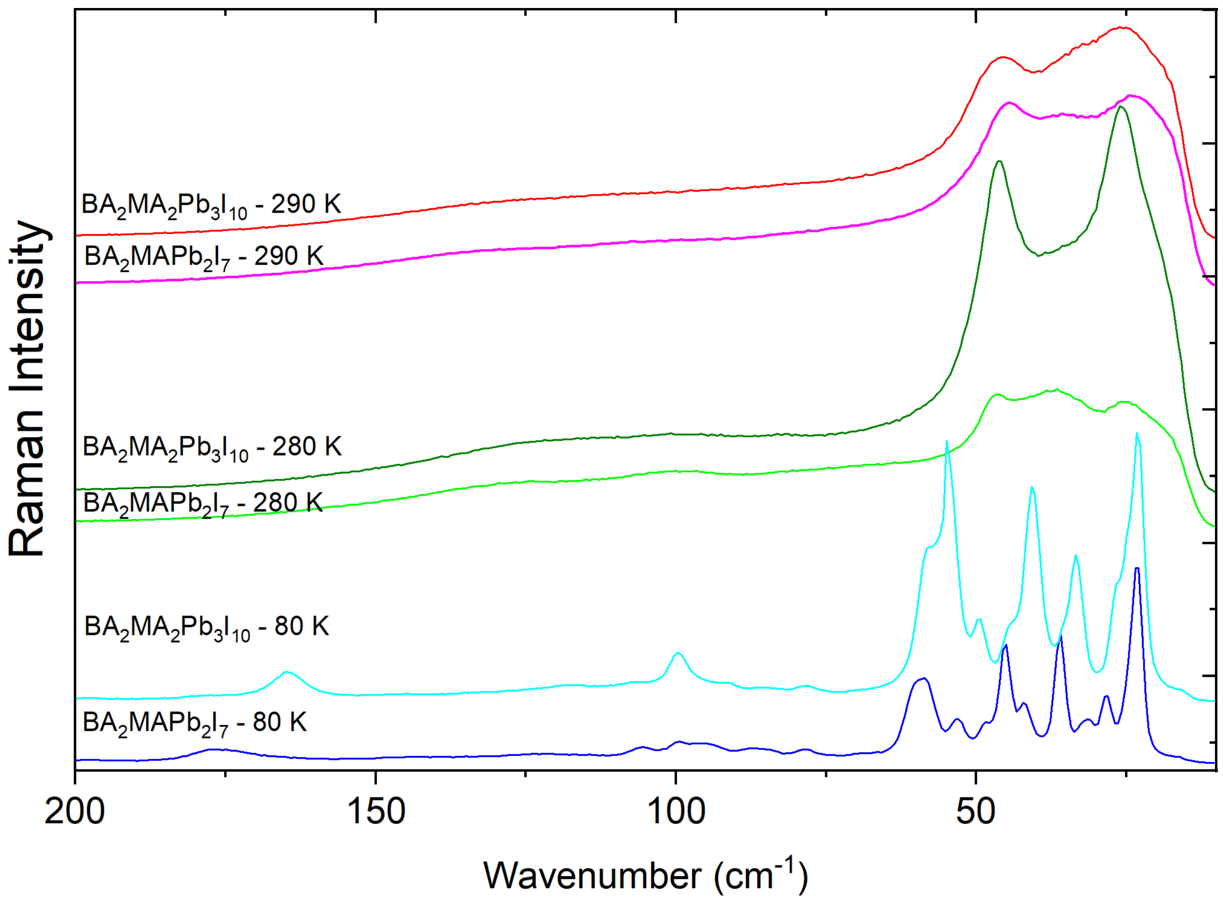

Figure S5. Comparison of Raman spectra of $BA_2MAPb_2I_7$ and $BA_2MA_2Pb_3I_{10}$ corresponding to three phases at 290, 280 and 80 K in the lattice modes region.

Table S1. Raman wavenumbers (in cm^{-1}) of $BA_2MAPb_2I_7$ at 360, 280 and 80 K together with the proposed assignment.[a] The bands above 300 cm^{-1} denoted in bold symbols correspond to internal modes of MA^+ .

360 K	280 K	80 K	assignment
1579s	1581s	1592w+1586w	**$\delta_{as}(NH_3^+)$**
	1571w	1570m	$\delta_{as}(NH_3^+)$
		1493vw	$\delta_s(NH_3^+)$
		1478vw	$\delta_s(NH_3^+)$
1473s	1475s	1467s	**$\delta_s(NH_3^+)$**
	1467s	1462s+1458sh	**$\delta_{as}(CH_3)$**+ $\delta_{as}(CH_3)$
1450m	1447m	1446w+1440w	$\delta_{as}(CH_3)$
1427w	1425w	1423w	$\delta_s(CH_3)$
1395vw	1394vw	1394w	$\omega(CH_2)+\delta_s(CH_3)$
	1379vw		$\omega(CH_2)$
		1363vw	$\tau(CH_2)$
1329w	1329w	1326w	$\tau(CH_2)$
	1314m	1315m	$\tau(CH_2)$
1289w	1292w	1291w	$\rho(CH_3)$
	1268w	1270w	$\rho(CH_3)$
1251vw	1257vw	1260w	**$\delta(CN)$**
1177sh	1182vw	1192vw	$\omega(CH_2)$
1157w	1163w	1164m	$\rho(NH_3^+)$
1073m	1074m	1075m	$\nu_{as}(CC)$
1040vw	1044w	1046w	$\nu_{as}(CC)$
1030vw	1032w		$\nu_{as}(CC)$
1002vw	999w	1010w	$\nu_{as}(CN)$
992vw	988m	1000m	$\nu_{as}(CN)$

963m	964m	972s	**ν(CN)**
	922w	929m+923vw	ω(NH,CH)
917s	914s	918vs	**$\rho(NH_3^+)+\rho(CH_3)$**
901m	900s	905s+900w	ω(NH,CH)
889m	892s	892m	ν_s(CC)
865m			ν_s(CN)
792w	796w+788s	794s	$\rho(CH_2)$
	736w	741w	$\rho(CH_2)$
476w	483w		δ_{as}(CCC)+δ_{as}(CCN)
416w	416m	419w	δ_s(CCN)
399w	398m	402w+399w	δ_s(CCC)
341w	335vw		??
236m	238m	240m	**MA-cage mode**
		196w	L+ T'+ Pb-I stretch
		176s	L+ T'+ Pb-I stretch
132sh	126s	143m+121m	L+ T'+ Pb-I stretch
104sh	99s	105s+100s+95s	L+ T'+ Pb-I stretch
		87m+85m+78m	L+ T'+ Pb-I stretch
44vs	47vs	61vs+58vs	Pb-I bend
		53m+48m+45vs	Pb-I bend
33vs	35vs	42m+36vs	Pb-I bend
22vs	23vs	32m+28m	$L(PbI_6)$
17sh	17sh	24sh+23vs	$L(PbI_6)$

[a] key: vs, very strong; s, strong; m, medium; w, weak; vw, very weak; sh, shoulder; ν, stretching; δ, bending (scissoring); ρ, rocking; ω, wagging; τ, twist; L, librational mode of organic cation; T', translational mode of organic cation.

Table S2. Raman wavenumbers (in cm^{-1}) of $BA_2MA_2Pb_3I_{10}$ at 320, 280 and 80 K together with the proposed assignment.[a] The bands above 300 cm^{-1} denoted in bold symbols correspond to internal modes of MA^+.

320 K	280 K	80 K	assignment
1579s	1579s	1593sh+1585m+1580sh	**$\delta_{as}(NH_3^+)$**
		1568m	$\delta_{as}(NH_3^+)$
1473m	1469vw	1466vs	**$\delta_s(NH_3^+)$**+ $\delta_{as}(CH_3)$
1453sh	1453sh	1453w+1446w+1440w	**$\delta_{as}(CH_3)$**
1426w	1425w	1423m	**$\delta_s(CH_3)$**
	1393vw	1395w	$\omega(CH_2)$
		1364vw	$\tau(CH_2)$
1324w	1329w	1326w	$\tau(CH_2)$
	1314w	1315m	$\tau(CH_2)$
		1291w	$\rho(CH_3)$
		1270w	$\rho(CH_3)$
	1254vw	1255w	**$\delta(CN)$**
		1195w+1190w	$\omega(CH_2)$
1157w	1162w	1164m	$\rho(NH_3^+)$
1073w	1073w	1075m	$\nu_{as}(CC)$
1035vw	1036vw	1045w	$\nu_{as}(CC)$
998vw	997vw	1010w+999m	$\nu_{as}(CN)$
963m	965m	970s	**$\nu(CN)$**
	923s	930w	$\omega(NH,CH)$
915m	914m	923s	**$\rho(NH_3^+)+\rho(CH_3)$**
900m	899m	905s+900sh	$\omega(NH,CH)$
890m	890m	892m	$\nu_s(CC)$
865w	863vw		$\nu_s(CN)$

790vw	789w	794m	ρ(CH_2)
		742w	ρ(CH_2)
473vw	483vw		δ_{as}(CCC)+δ_{as}(CCN)
415vw	415vw	419w	δ_s(CCN)
	398vw	404w+399w	δ_s(CCC)
		307w	??
236m	234m	237m	**MA-cage mode**
		198w	L+ T'+ Pb-I stretch
		178w	L+ T'+ Pb-I stretch
		164m	L+ T'+ Pb-I stretch
127s	123s	118m	L+ T'+ Pb-I stretch
100s	99s	106w+100s+92w	L+ T'+ Pb-I stretch
	82w	85w+78w	Pb-I bend
45vs	47vs	58s+54vs	Pb-I bend
		49m+45m	Pb-I bend
32s	33s	41vs	Pb-I bend
22vs	25vs	33s	L(PbI_6)
17sh	19sh	26sh+23vs	L(PbI_6)

[a] key: vs, very strong; s, strong; m, medium; w, weak; vw, very weak; sh, shoulder; ν, stretching; δ, bending (scissoring); ρ, rocking; ω, wagging; τ, twist; L, librational mode of organic cation; T', translational mode of organic cation.
